# Cardiovascular and thermal strain during 3–4 days of a metabolically demanding cold-weather military operation

**DOI:** 10.1186/s13728-017-0056-6

**Published:** 2017-09-06

**Authors:** John W. Castellani, Marissa G. Spitz, Anthony J. Karis, Svein Martini, Andrew J. Young, Lee M. Margolis, J. Phillip Karl, Nancy E. Murphy, Xiaojiang Xu, Scott J. Montain, Jamie A. Bohn, Hilde K. Teien, Pål H. Stenberg, Yngvar Gundersen, Stefan M. Pasiakos

**Affiliations:** 1 United States Army Research Institute of Environmental Medicine, 10 General Greene Avenue, Bldg 42, Natick, MA 01760 USA; 2 Norwegian Defence Research Establishment, Kjeller, Norway; 3 General Defence Material/Catering and Combat Feeding Section, Norwegian Navy, Rodskferveien, Norway

**Keywords:** Deep body temperature, Finger temperature, Heart rate, IREQ, Thermal modeling

## Abstract

**Background:**

Cardiovascular (CV) and thermal responses to metabolically demanding multi-day military operations in extreme cold-weather environments are not well described. Characterization of these operations will provide greater insights into possible performance capabilities and cold injury risk.

**Methods:**

Soldiers from two cold-weather field training exercises (FTX) were studied during 3-day (study 1, *n* = 18, age: 20 ± 1 year, height: 182 ± 7 cm, mass: 82 ± 9 kg) and 4-day (study 2, *n* = 10, age: 20 ± 1 year, height: 182 ± 6 cm, mass: 80.7 ± 8.3 kg) ski marches in the Arctic. Ambient temperature ranged from −18 to −4 °C during both studies. Total daily energy expenditure (TDEE, from doubly labeled water), heart rate (HR), deep body (*T*_pill_), and torso (*T*_torso_) skin temperature (obtained in studies 1 and 2) as well as finger (*T*_fing_), toe (*T*_toe_), wrist, and calf temperatures (study 2) were measured.

**Results:**

TDEE was 6821 ± 578 kcal day^−1^ and 6394 ± 544 for study 1 and study 2, respectively. Mean HR ranged from 120 to 140 bpm and mean *T*_pill_ ranged between 37.5 and 38.0 °C during skiing in both studies. At rest, mean *T*_pill_ ranged from 36.0 to 36.5 °C, (lowest value recorded was 35.5 °C). Mean *T*_fing_ ranged from 32 to 35 °C during exercise and dropped to 15 °C during rest, with some *T*_fing_ values as low as 6–10 °C. T_toe_ was above 30 °C during skiing but dropped to 15–20 °C during rest.

**Conclusions:**

Daily energy expenditures were among the highest observed for a military training exercise, with moderate exercise intensity levels (~65% age-predicted maximal HR) observed. The short-term cold-weather training did not elicit high CV and *T*_pill_ strain. *T*_fing_ and *T*_toe_ were also well maintained while skiing, but decreased to values associated with thermal discomfort at rest.

## Background

Warfighters can expend more energy (~10–40%) during cold-weather operations due to heavy clothing, carrying extra weight, and the effort to work/walk/ski in snow and difficult terrain [1–3]. This may accelerate the development of fatigue, reducing operational effectiveness and performance [4]. Exercising in the cold to fatigue can also lead to thermal strain, as evidenced by a decline in core temperature [5–7]. Furthermore, cold-weather operations may reduce peripheral toe and finger skin temperatures [8, 9], primarily after stopping work, increasing susceptibility to cold injury and decreasing dexterity and tactile sensation.

Higher exercise intensities can lower thermal strain in the cold by causing higher deep body and peripheral temperatures. Laboratory studies clearly demonstrate this in cold-wet [10, 11] and cold-dry [12] conditions. Similar findings are observed in field and occupational settings [9, 13, 14]. For example, during light, moderate, and heavy exercise intensities over 6-h work days [13], rectal temperature was lowest during light work (~275 W) and highest during work eliciting ~435 W and finger (*T*_fing_) and toe (*T*_toe_) temperatures followed the same pattern. These field studies cited were conducted during limited cold exposure, that is, exposure durations less than 6 to 8 h over a 24-h period with access to heated shelter when not working. Similarly, adventure sporting events (e.g., Iditarod Trail Invitational, 6633 Ultra), can take place over multiple days under extreme environmental conditions where participants self-select their exercise intensity. Furthermore, these military and sporting scenarios often have multiple exacerbating physiological stressors, including very high energy expenditures and energy deficits [15]. This cumulative exposure to cold and other stressors may increase susceptibility to cold injuries [16], but the impact of multiple days of sub-freezing cold exposure during field training on cardiovascular and thermal strain during high deficits are not well described.

Cold-weather guidance and thermoregulatory models are available to determine the clothing insulation required (IREQ) during cold-weather work to maintain thermal balance [17, 18] and to predict deep body and skin temperatures during cold exposure (e.g., 6-cylinder (6-CTM) model developed by Xu et al. [19, 20]). Although these models have been available for over two decades, validation studies have been limited. Previous occupational studies using the IREQ to determine if adequate clothing was used by workers and soldiers during cold exposure has only been studied over the course of one working day [21–23]. Thermoregulatory models for use in cold conditions have primarily been validated and tested during cold-water immersion [24, 25] and not during exercise in cold air.

The purpose of this study was to characterize the cardiovascular and thermal strain experienced by Soldiers with significant energy deficits participating in two winter military field-training exercises in the Norwegian Arctic [26, 27]. A secondary purpose was to determine the insulation required (IREQ) to maintain thermal balance during these winter exercises and determine, post hoc, if this level of insulation was sufficient for the multiple day winter exercise, as well as compare measured to predicted values using the 6-CTM. We hypothesized that cardiovascular strain would be moderate and that deep body thermal strain would not reach hypothermic levels due to proper clothing access and adherence to risk management strategies. We also hypothesized that the clothing required to maintain thermal balance during multiple days of Arctic ski marching would be adequate.

## Methods

### Subjects and experimental design

#### Study 1

Eighteen male Norwegian Soldiers [age: 20 ± 1 year, height: 182 ± 7 cm, mass: 82 ± 9 kg, (mean ± SD)] volunteered after providing informed, written consent [26]. This study was approved by the Institutional Review Board at the US Army Research Institute of Environmental Medicine (Natick, MA, USA) and the Regional Committees for Medical and Health Research Ethics (Oslo, NO).

The Soldiers participated in a 7-day winter training exercise that culminated with a 3-day 54-km cross-country ski march in the Norwegian Arctic during mid-March. During the March, the volunteers skied in 50:10 min work-to-rest ratios for 6–10 h per day. Each volunteer carried/wore ~45 kg of equipment throughout the ski march. The ski march pace was self-selected. At the end of each day, the Soldiers set up and slept in 10-man Arctic tents. Four Norwegian military cold-weather rations, (~5100 kcal day^−1^ if entirely consumed) were provided each day. Data are reported on the 3-day ski march portion of the training exercise only.

#### Study 2

Ten male Norwegian Soldiers (age: 20 ± 1 year, height: 182 ± 6 cm, mass: 80.7 ± 8.3 kg) volunteered for study 2 after providing informed, written consent. The volunteers in study 2 were a different cohort than the volunteers in study 1. These Soldiers were a subset of the total number of Soldiers who volunteered for a larger study (*n* = 73, [27]). This study was approved by the Institutional Review Board at the US Army Research Institute of Environmental Medicine (Natick, MA, USA) and the Regional Committees for Medical and Health Research Ethics (Oslo, NO).

Similar to study 1, the Soldiers skied, self-paced, in 50:10 min work-to-rest ratios, while carrying a 45-kg pack, over a total distance of 51 kM, in the Norwegian Arctic in late January. The Soldiers slept in one- to four-man tents and were responsible for digging and setting up their tent site. This study differed from study 1 with regard to food allocated to each volunteer as these volunteers received only three Norwegian cold-weather rations per day (~3500 kcal day^−1^ if entirely consumed). The volunteers were further randomized to one of three feeding groups, protein (PRO) supplementation, carbohydrate (CHO) supplementation, and control (CON, no supplementation). In the PRO and CHO groups, the supplement provided an additional 1000 kcal day^−1^. Of the 10 volunteers in the thermal strain group, there were 5 in the CHO group, 2 in the PRO group, and 3 in the CON group. The data for these three feeding groups were pooled for the analyses reported in this paper as there were no differences for the groups.

### Measurements

Volunteers wore a physiological monitoring system (PSM; Equivital-1, Hidalgo Ltd. Cambridge, UK) to measure heart rate (HR), chest skin temperature (*T*_chest_), and core temperature (*T*_pill_) in both studies. The HR and *T*_chest_ sensors were imbedded within the chest strap and shoulder support of the PSM. Ingested temperature telemetry pills (Jonah™ Core Temperature Pill, Respironics, Bend OR) were used to obtain *T*_pill_.

In study 2, HR, *T*_chest_, and *T*_pill_ were measured as in study 1. Additional skin temperature measurements were obtained on the wrist (ventral, non-dominant hand), 3rd finger (nailbed), right gastrocnemius, and right 1st toe (nailbed). Wrist and calf temperatures were obtained using iButton data loggers (Dallas Semiconductor, Dallas, TX) affixed with Velcro straps; finger and toe temperatures were measured using thermistors affixed to the nailbed region using an adhesive and secondarily secured with medical grade tape.

Energy expenditure (EE) and energy intake (EI) were measured using doubly labeled water and subject self-report of items eaten, respectively. These measurements and values are reported in detail elsewhere [26, 27]. Table 1 shows the EE, EI, and energy deficit for studies 1 (*n* = 17) and 2 (*n* = 9).

**Table 1:** Energy expenditure, intake, and balance during study iterations #1 and #2

	Energy expenditure	Energy intake	Energy balance
Study 1 (*n* = 17)	6821 ± 578 (28.5 ± 2.4)	3465 ± 622 (14.5 ± 2.6)	−3357 ± 691 (−14.0 ± 2.9)
Study 2 (*n* = 9)	6394 ± 544 (26.8 ± 2.3)	2714 ± 799 (11.4 ± 3.3)	−3782 ± 1001 (−15.8 ± 4.2)

Values (mean ± S.D.) are in kcals and MJ (parenthetically)

Weather data were obtained from the closest meteorological station to each training exercise by accessing the Norwegian Meteorological Institute website (http://eklima.met.no). In study 1, air temperatures ranged from −18 to −6 °C; wind speeds ranged from 5 to 13 m s^−1^; in study 2, air temperatures ranged from −17 to −3 °C and wind speeds ranged from 0.3 to 19 m s^−1^. These environmental conditions produced wind chill temperature index values as low as −26 °C in study 1 and −25 °C in study 2.

In both winter training exercises, the Soldiers wore the same uniform. Clothing insulation over the torso, arms, and legs was ~2.75 clo and consisted of a woolen mesh base undergarment, wool middle layer, and waterproof/windproof outer layer. Foot insulation, provided by a wool sock and cold-weather boot, was 1.76 clo. Thermal (Rt, m^2^ C W^−1^) and evaporative resistance (Re, m^2^ kPa W^−1^) was determined using a thermal manikin (TM) following the standard outlined in ASTM 1291-10. Briefly, the manikin mean skin temperature was set at 35 °C, with the ambient temperature and air velocity set at 23 °C and 0.4 m s^−1^, respectively. The TM was dressed in the Norwegian military cold-weather uniform worn during the ski marches, consisting of a base, mid-, and outer layer.

The insulation required (IREQ) and duration limited exposure (DLE) during ski marching on day 2 of Study 2 were calculated (Lund University website—http://www.eat.lth.se) according to ISO 11079 [18] and based upon the work of Holmer [17]. The assumptions for calculating IREQ and DLE were as follows: predicted metabolic rate [28] for a walking speed of 0.66 m s^−1^, terrain coefficient of 1.7, and external load of 50.9 kg. This value for metabolic rate was increased by an additional 40% due to the layering effect of cold-weather clothing and the foot mass effect [29–32]. As well, mean air temperature and wind speed measured during the movement portion of Day 2 was input. Day 2 from study 2 was chosen for modeling since the day 1 movement was shorter in duration and a large storm came in on day 3. As well, we did not obtain skinfold measurements during study 1 and thus could not estimate % body fat.

We also determined the goodness of fit between observed temperatures (pill, chest, calf) and the 6-CTM [19, 33]. The 6-CTM model was derived from 2 previous thermoregulatory models [34, 35]. The 6-CTM describes the human body as a passive system of six cylinders: head, trunk, arms, legs, hands, and feet. Each cylinder is further concentrically divided into compartments representing the core, muscle, fat, and skin. Blood is represented as a one-loop circulatory system and is an independent compartment. Thus, the human body is represented by 25 compartments. The sizes of the compartments are determined from height, weight, and body fat percentage [19, 33]. The 6-CTM inputs include individual characteristics (i.e., height, weight, fat percentage, age, VO_2max_), predicted external workload [28] for walking at 0.66 m s^−1^ using a terrain coefficient of 1.7, and environmental (i.e., temperature, humidity, and wind velocity) and clothing (clothing insulation clo, moisture permeability index *i*_*m*_) parameters for each of the six cylinders. The model used in this analysis uses a standard initial starting core temperature of 36.8 °C. For *T*_pill_, we adjusted the model predictions by using changes in pill temperature rather than absolute temperatures so that we could compare between observed and predicted values. Comparisons (measured vs. predicted, respectively) were made between *T*_pill_ and *T*_core_, *T*_chest_ and *T*_torso_, and *T*_calf_ and *T*_leg_. *T*_wrist_ was not compared to the model as there is no corresponding site in the 6-CTM.

### Statistical analysis

Differences between observed temperatures and 6-CTM model predictions were evaluated by comparing the root mean square deviation (RMSD) of each trial with the observed standard deviation [36]. This statistic is used to quantitatively determine the goodness-of-fit between model predictions and observed data. The RMSD (°C) is defined as

$${\text{RMSD }} = \sqrt {\frac{1}{n}} \sum\limits_{i = 1}^{n} {d_{i}^{2} },$$

where d_i_ is the difference between observed and predicted core temperature response at each time point (°C) and *n* is the number of time points examined with an interval of 10-min used. The prediction was considered valid if the RMSD fell below the SD of the observed values [33].

Non-parametric Bland–Altman plots were also used to determine the level of agreement [37] between the observed and predicted temperatures for the 6-CTM using ±0.4 °C as a qualitative physiological threshold for assessment for *T*_pill_ and ±1.0 °C for skin temperature. This threshold is twice the anticipated standard deviation for core temperature [38] which accounts for unique and additive response variability likely to occur with experimental perturbations. Smaller differences are therefore within the acceptable noise of the measurement and were considered of marginal importance independent of the *p* value. This procedure allows data evaluation against an evidentiary standard other than zero, similar to equivalence testing [39]. Data for Bland–Altman plots were binned into three 3-h time blocks to examine differences between observed and predicted values over time.

## Results

### Heart rate, pill, and skin temperatures

#### Study 1

**Fig. 1:**
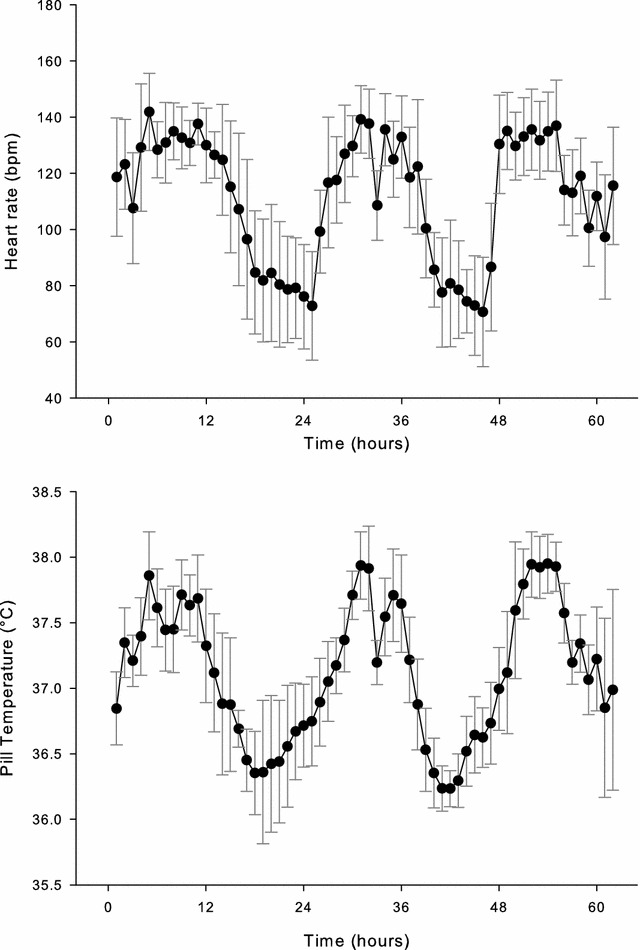
Heart rate (HR) and pill temperature (*T*_pill_) vs. time during 3-day Arctic ski march (study 1). Data are mean ± S.D. (HR: *n* = 18; *T*_pill_: *n* = 10)

Figure 1 shows the HR response, over 1-h periods, during the first training exercise (*n* = 18). During the physical activity portion of each day, the mean heart rate was 130 ± 2 beats per min (bpm), or ~65% age-predicted maximal heart rate. Between daily bouts of ski marching, the HR averaged ~88 bpm, with values between 70 and 80 bpm during sleep. During the prolonged periods of skiing, the mean *T*_chest_ was 34.1 ± 0.7 °C (*n* = 18), with a slight elevation during the rest periods (35.5 °C). Figure 1 also shows the *T*_pill_ response every hour over the course of the ski march (*n* = 10). *T*_pill_ averaged 37.5 ± 0.1 °C during the exercise periods, reaching a nadir (36.27 °C) ~5 h into each rest period. The mean maximal *T*_pill_ (*n* = 10) during skiing was 38.2 ± 0.2 °C (highest value = 38.4 °C). At rest, the mean minimal *T*_pill_ was 36.0 ± 0.3 (lowest value = 35.5 °C).

#### Study 2

**Fig. 2:**
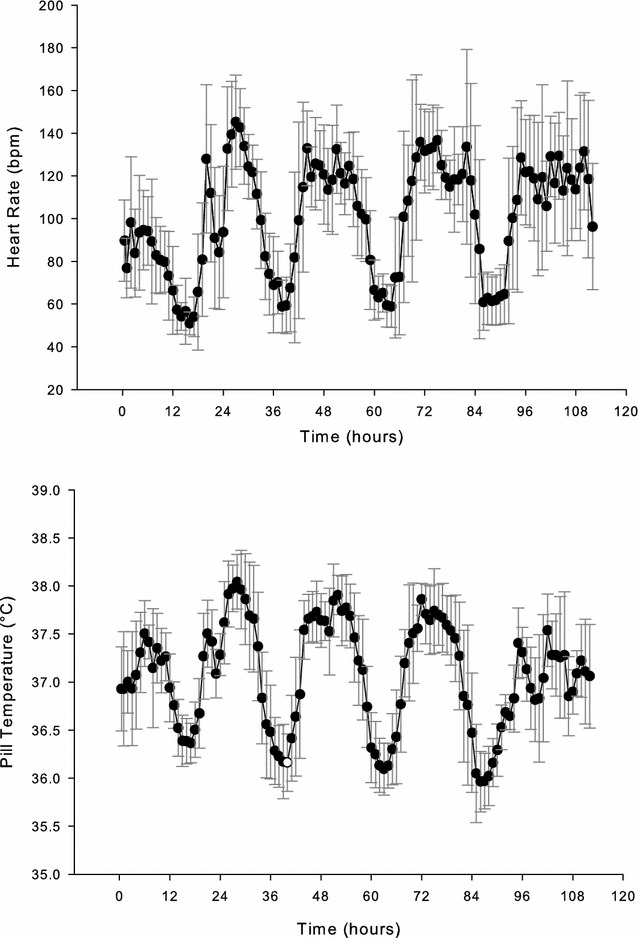
Heart rate and pill temperature vs. time during 4-day Arctic ski march (study 2, *n* = 10). Data are mean ± S.D

The HR response every hour over the study period is depicted in Fig. 2 (*n* = 10). The overall HR was highest for the first day of the ski march and bivouac (~140 bpm). During subsequent days, the HR was ~120–130 bpm, similar to that observed in study 1. During the rest periods, HR decreased to 60 bpm.

Figure 2 shows the *T*_pill_ response over the training period (*n* = 10). The mean *T*_pill_ was highest during the first movement, paralleling that of HR. The highest mean *T*_pill_ during this first movement was 38.07 °C. During the rest periods, the average *T*_pill_ fell to ~36.0–36.2 °C, with the lowest values observed on the third night, when the soldiers were confined to their tents due to a storm. The lowest individual *T*_pill_ during this time was 35.6 °C. *T*_chest_ values ranged between 33 and 36 °C, with the highest values obtained when the volunteers were resting in their tents.

**Fig. 3:**
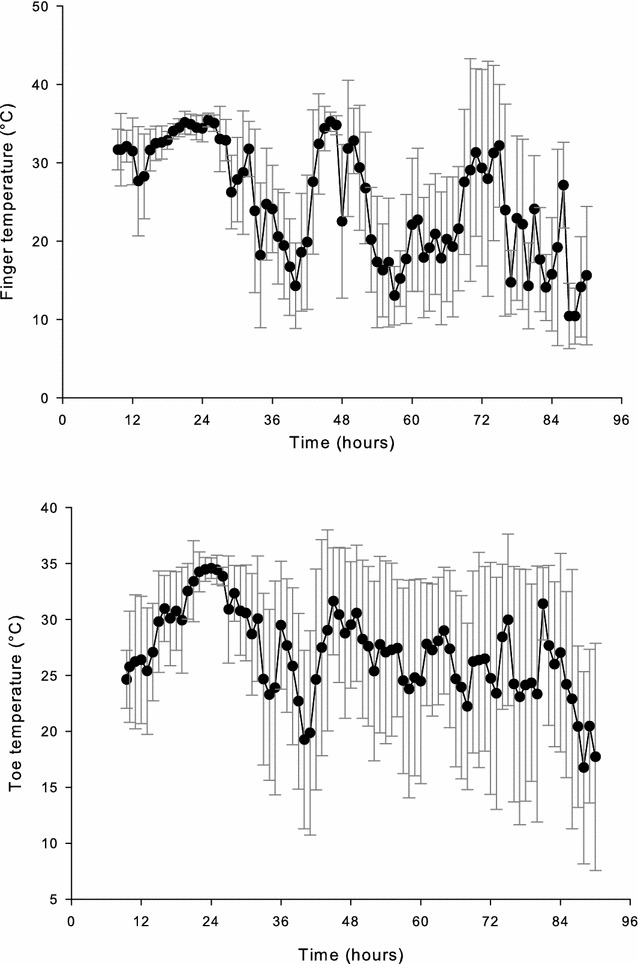
Finger (*T*_finger_) and toe (*T*_toe_) temperatures vs. time during 4-day Arctic ski march (study 2). Data are mean ± S.D. (*T*_finger_: *n* = 9; *T*_toe_: *n* = 10)

Peripheral skin temperatures [*T*_fing_ (*n* = 9) and *T*_toe_ (*n* = 10)] are shown in Fig. 3. During the first 2 days of ski marching, *T*_fing_ reached as high as 35 °C. Over the last 2 days of the March, *T*_fing_ ranged between 25 and 30 °C. During the rest periods, *T*_fing_ was typically between 15 and 20 °C, although during the storm, temperatures fell as low as 6–10 °C. Similar to *T*_fing_, *T*_toe_ reached 35 °C during the first movement. Subsequent *T*_toe_ during the ski marches was between 25 and 30 °C. During the rest periods, *T*_toe_ was between 15 and 20 °C. *T*_wrist_ (*n* = 5) during exercise was ~33 °C; at rest *T*_wrist_ was 25–26 °C. *T*_calf_ (*n* = 4) fluctuated between 30 and 35 °C throughout the training exercise.

### IREQ and 6-CTM predictive model

During the 2nd day of ski marching in the second study, the calculated IREQ required to maintain thermal balance at a normal mean body temperature ranged from 2.0 to 2.6 clo; the DLE was calculated to be greater than 8 h.

**Fig. 4:**
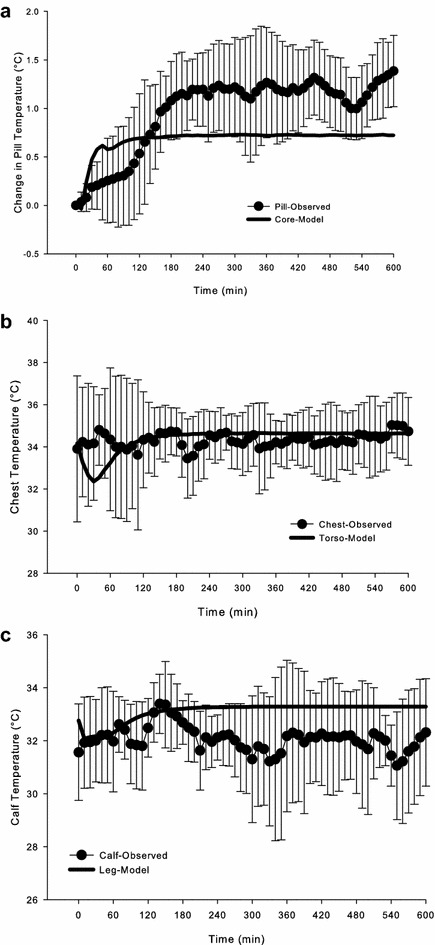
Observed (mean ± S.D.) and predicted change in pill (**a**), chest (**b**), and calf (**c**) temperatures vs. time during 10-h ski march in −6.1 °C air temperature. Predicted values were calculated using the 6-CTM thermoregulatory model

Figure 4 shows the observed values over the 10-h time period compared to the predicted values using the 6-CTM. The SD and RMSD for each trial are presented in Table 2.

**Table 2:** Standard deviations and root mean square deviations (RMSD) for 3 temperature sites during 10-h ski march (hours 41–51) in Study 2

Measurement	No. of volunteers	Standard deviation	RMSD
Pill (Core)	10	0.45	0.59
Chest (Torso)	8	1.67	1.34
Calf (Leg)	4	1.70	1.89

Sites in parentheses denote site for model prediction

**Fig. 5:**
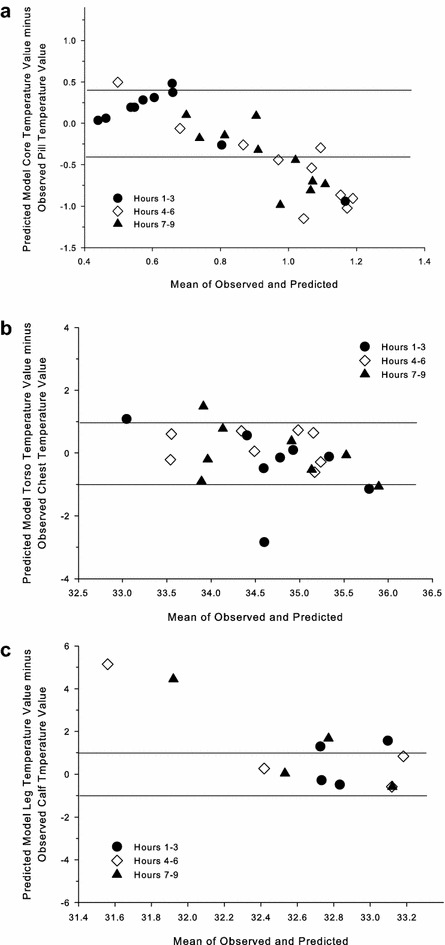
Bland-Altman plots for the change in change in pill (**a**), chest (**b**), and calf (**c**) temperatures using the 6-CTM thermoregulatory model. *Lines* shown are the qualitative thresholds (±0.4 °C for pill temperature; ±1.0 °C for skin temperatures) for prediction agreement

Using the RMSD criterion, the 6-CTM fits the data for chest temperature, but RMSD was higher than the SD for *T*_pill_ and *T*_calf_. To better evaluate the practical importance of the differences between predicted and measured values, non-parametric Bland–Altman plots were constructed for each trial to determine what percentage of the predicted values fell within a qualitative threshold of importance (0.4 °C for *T*_pill_ and 1.0 °C for skin temperatures). Figure 5 presents these data over time. For *T*_pill_, 53% of the observations fell between ±0.4 °C, with the model under-predicting the observed values as time elapsed. For chest temperature, 79% of the values were between ±1.0 °C, in agreement with the RMSD value, which was lower than the SD. For calf temperature, 58% of the values were between ±1.0 °C. Two values were clearly over-predicted; these were from the same volunteer.

## Discussion

This study showed that 3–4 days of ski marching in below freezing temperatures elicited only mild cardiovascular and thermal strain, suggesting that the Soldiers’ clothing and equipment provided good protection under the environmental and physical conditions studied. Heart rates during skiing averaged ~130 beats min^−1^ suggesting that Soldiers worked at ~45% VO_2max_ for 6–10 h per day. Deep body temperature, measured with *T*_pill_, reflects this moderate exercise intensity, with values ranging from 37.5 to 38.0 °C. However, during some periods of study 2, finger temperatures as low as 6–10 °C were observed during the rest periods, values well below the discomfort (20 °C) and manual dexterity (15 °C) threshold [4, 40, 41]. Likewise, toe temperatures fell to 15–20 °C, well above any potential freezing cold injury risk, but cold enough to elicit discomfort. It is likely that during these periods of low finger and toe temperatures the Soldiers removed their gloves and boots/socks.

The degree of physiological strain is similar to hikes/military movements conducted over a 3–7.5 h timeline while carrying external masses of 9.5–31 kg [23, 42, 43]. In these “like” studies, HRs ranged from 110 to 135 beats min^−1^ (55–73% HR_max_), similar to that observed in the present study. The self-selected exercise intensities (~35 VO_2max_) in our study were also consistent with a 6-day military exercise [44] during which Soldiers carried external mass. Interestingly, this exercise intensity of 35–50% VO_2max_ exhibited with load carriage is comparable to reports for self-paced or self-selected exercise in temperate conditions without carrying external mass. Ekkekakis [45] summarized the literature for self-selected exercise intensity and showed in the majority of studies across a wide spectrum of exercise and activities of daily living (running, walking, lawn mowing, house cleaning) that people voluntarily choose to exercise between 60–70% HR_max_ and 40–60% VO_2max_. Adventure races also provide insight into exercise intensities; studies demonstrate that participants in these multi-day events choose intensities at ~40% VO_2max_ [46, 47].

Deep body temperatures (*T*_pill_) throughout the ski marches were unremarkable, and similar to other occupational scenarios in a variety of environmental conditions. Virokannas [13] presented histograms of core temperature during outside work comprising light, moderate, and heavy work in cold environmental conditions (−10 to 5 °C). He found that during moderate work (340 W), rectal temperature (*T*_re_) was primarily distributed around 37.5 °C, while heavy work (420 W) caused *T*_re_ to primarily range between 37.5 and 38 °C. Ainslie et al. [42] observed *T*_re_ values between 37.5 and 38 °C during 7.5 h of hill walking (9.5 kg external load carriage) when consuming 3000 kcal (about a 1700 kcal deficit). Similarly, Morabito et al. [23] reported pill temperatures of ~38 °C after a 12-km winter movement in 3 h, about a 1 °C increase from rest. Sleeping deep body temperatures in the current study were similar to that observed during a 54-h physically intense, energy intake-restricted field training exercise in warmer ambient conditions [15], indicating that the insulation from clothing, sleeping bags, and shelter was adequate to protect against freezing injury during the two winter training exercises.

Peripheral skin temperatures, as would be expected from laboratory studies, changed as a function of exercise and rest cycles [12, 48, 49], with the highest values occurring during activity and decreasing during rest, occasionally falling below the dexterity performance threshold (15 °C). Similar findings have also been observed during military field training exercises. *T*_toe_ during guard duty (rest) were 15.4 °C and increased to 31.2 °C during hiking at an O_2_ uptake of 1–1.5 L/min [9]. Similarly, Rissanen et al. [8] showed during a 12-day field exercise (*T*_air_: −20 to 2 °C), combat activities eliciting a HR of ~102 bpm raised *T*_fing_ to 23.1 °C, compared to a *T*_fing_ of 19.8 °C when HR was 89 bpm. Unlike in the Mejkavic study, there were no differences in *T*_toe_ (28.8 °C) between activity levels. In quite disparate occupational settings (railyard workers, surveying, construction, patrol skiing) in subarctic Finland [50], finger temperatures averaged 20–28 °C, with minimum values of 6–10 °C, similar to the observations in the current study.

The calculated IREQ values ranged from 2.0 to 2.6 clo. This was due to the moderate metabolic heat production values (~400 W) caused by skiing. Thus, the insulation provided by the cold-weather uniform (2.75 clo) was more than adequate to prevent a fall in deep body temperature. Indeed, during the ski marches, pill temperature increased to mean values of 37.5–38 °C throughout the long exercise period. The DLE reflected these deep body temperatures by calculating work limits that extended beyond 8 h. Morabito et al. [23] completed an extensive evaluation of the IREQ during a winter military exercise (12-km hike in 3 h). They found that the estimated neutral IREQ during the hike was ~50% lower (1.6 clo) in 5 out of the 6 soldiers than what was worn (3 clo). Not surprisingly deep body temperature increased by 1 °C. A field validation study concluded that the IREQ does not predict needed clothing insulation as well during relatively high-intensity work [51]. Although the required insulation was adequate in the current and previous studies, one concern is that the increase in deep body temperature can stimulate sweating, and if clothing layers are not adequately managed, can potentially compromise insulation. One limitation of IREQ/DLE is that predicted peripheral temperatures are not included in determining risk susceptibility or work time. Adding thermal models that predict finger/toe temperatures [52, 53] to the IREQ would provide useful information for determining overall cold injury risk susceptibility and enable greater precision for cold-weather guidance.

Comparisons between observed values for *T*_pill_, *T*_chest_, and *T*_calf_ and predicted values for *T*_core_, *T*_torso_, and *T*_leg_, respectively, using the 6-CTM are one of the first reported comparisons made during exercise in cold air at temperatures below freezing; a recent paper compared the Fiala thermophysiological model to values obtained during walking at 60% VO_2peak_ in 10 °C air [54]. Observed *T*_chest_ values were best fit by the 6-CTM, whereas both *T*_pill_ and *T*_calf_ had RMSD values above the SD and 50–60% of the values within the accepted criterion using non-parametric Bland–Altman plots. Core temperature was predicted within the SD and the 0.4 °C criterion for the first 3 h of exercise in −6.2 °C air (wind chill temperature index of −14 °C), but under-predicted during the rest of the ski march for Day 2. Predicted *T*_calf_, although with a higher RMSD than S.D., was within the measured SD (Fig. 4). A limitation in the present study is the estimation of metabolic rate from prediction equations; measuring oxygen uptake during these field conditions would enable more accurate validation of thermal models during exercise in cold air. As well, model validation in these conditions will allow better predictions of the effects of clothing on core and skin temperatures. Similar to the IREQ, a limitation of the 6-CTM is that it does not predict peripheral finger and toe temperatures. This addition to the thermal model would enable it to be used for predicting the peripheral areas most susceptible to freezing cold injury for use in developing cold-weather guidance.

## Conclusions

In summary, 3–4 days of ski marching during winter field training exercises in the Arctic elicits moderate cardiovascular and thermal (deep body and torso skin temperature) strain when daily energy intake is 3300–3800 kcals lower than daily energy expenditure. The greatest risk to Soldiers during these types of military exercises is a decrease in peripheral temperatures (finger, toe) that can possibly impair manual dexterity and increase thermal discomfort levels. These characterizations of cardiovascular and thermal strain during military cold-weather operations allow for improved recommendations to be made in an attempt to mitigate potential cold injury risk that could compromise physical performance and military readiness. Predictive thermal modeling demonstrated moderate agreement between observed and predicted values for deep body and skin temperatures. Adding finger and toe temperature predictive capabilities to the IREQ and 6-CTM would increase their ability for providing guidance for cold injury risk prevention.

## Abbreviations

6-CTM: 6-cylinder thermoregulatory model
CHO: carbohydrate supplementation group
CON: control group
DLE: duration limited exposure
EE: energy expenditure
EI: energy intake
HR: heart rate
IREQ: insulation required
PRO: protein supplementation group
RMSD: root mean square deviation
SD: standard deviation
T_calf_: calf skin temperature
T_chest_: chest skin temperature
T_fing_: finger skin temperature
T_pill_: gastrointestinal pill temperature
T_toe_: toe skin temperature
T_re_: rectal temperature
T_wrist_: wrist skin temperature
VO_2max_: maximal oxygen uptake
